# Studies with Human-Induced Pluripotent Stem Cells Reveal That CTNS Mutations Can Alter Renal Proximal Tubule Differentiation

**DOI:** 10.3390/ijms242317004

**Published:** 2023-11-30

**Authors:** Ramkumar Thiyagarajan, Mary Taub

**Affiliations:** 1 Division of Geriatric Medicine, University of Kansas Medical Center, University of Kansas, Kansas City, KS 66160, USA; rthiyagarajan@kumc.edu; 2 Biochemistry Department, Jacobs School of Medicine and Biomedical Sciences, University at Buffalo, Buffalo, NY 14203, USA

**Keywords:** cystinosis, cystinosin, renal proximal tubule, hiPSCs, lysosomal cystine transporter, differentiation, tubulogenesis

## Abstract

Cystinosis is an autosomal recessive disease resulting from mutations in *ctns*, which encodes for cystinosin, a proton-coupled cystine transporter that exports cystine from lysosomes. The major clinical form, infantile cystinosis, is associated with renal failure due to the malfunctioning of the renal proximal tubule (RPT). To examine the hypothesis that the malfunctioning of the cystinotic RPT arises from defective differentiation, human-induced pluripotent stem cells (hiPSCs) were generated from human dermal fibroblasts from an individual with infantile cystinosis, as well as a normal individual. The results indicate that both the cystinotic and normal hiPSCs are pluripotent and can form embryoid bodies (EBs) with the three primordial germ layers. When the normal hiPSCs were subjected to a differentiation regime that induces RPT formation, organoids containing tubules with lumens emerged that expressed distinctive RPT proteins, including villin, the Na^+^/H^+^ Exchanger (NHE) isoform 3 (NHE3), and the NHE Regulatory Factor 1 (NHERF1). The formation of tubules with lumens was less pronounced in organoids derived from cystinotic hiPSCs, although the organoids expressed villin, NHE3, and NHERF1. These observations can be attributed to an impairment in differentiation and/or by other defects which cause cystinotic RPTs to have an increased propensity to undergo apoptosis or other types of programmed cell death.

## 1. Introduction

Cystinosis is an autosomal recessive disease resulting from mutations in the *ctns* gene that encodes for cystinosin [1], a member of the Lysosomal Cystine Transporter (LCT) family, with significant homology to Microbial Rhodopsins (MRs) [2]. Cystinosin is a cystine/proton cotransporter that extrudes cystine from lysosomes [1]. However, cystine efflux from lysosomes declines, as a consequence of a number of the *ctns* mutations that alter cystinosin, and as a consequence, intracellular cystine levels increase to such an extent that cystine crystals form [3]. Cystinosis has three major clinical forms, based on the age of onset as well as the severity of the disease [3]. They include infantile cystinosis, with an early onset, juvenile cystinosis, with a late onset, and non-nephropathic cystinosis. Infantile cystinosis (also called nephropathic cystinosis), the most severe form of cystinosis, affects 95% of afflicted individuals, being detected as early as 6 months of age, due to the appearance of the Fanconi Syndrome. Excessive levels of such solutes as sodium, phosphate, bicarbonate, amino acids, and glucose appear in the urine of infants with the Fanconi Syndrome due to the defective reabsorption by the Renal Proximal Tubule (RPT). Consequences include rickets, growth retardation, and end-stage renal failure by around 10 years of age [3]. Subsequently, other organs are affected. Although cystinosis occurs at a low frequency (1 in 100,000 to 200,000 births), the consequences of the disease are devastating, drastically limiting the patient’s lifespan even when administering therapy (which is expensive).

The most common form of therapy is the administration of cysteamine [4], a drug that reacts with lysosomal cystine, yielding cysteine and a cysteamine-cystine disulfide, both of which leave the lysosomes, thereby reducing lysosomal cystine levels. Although cysteamine increases the lifespan, the effects of cysteamine are only transient (requiring ingestion every 6 h). In addition, the drug is costly, and there are problems with compliance due to complications caused by the drug (including skin rashes, ulcers, blurry vision, and bad breath). Moreover, cysteamine does not prevent the Fanconi syndrome [5]. Unfortunately, alternative treatment options are not available. Thus, it is imperative that a further understanding of the molecular consequences of elevated lysosomal cystine is obtained.

In vitro studies utilizing tissue culture models of cystinosis are extremely important in these regards. However, initially, in vitro studies were seriously limited, being conducted with undifferentiated, cystinotic fibroblasts [6]. Although increased cystine levels are observed in all cells in the body in cystinosis, only specific tissues are affected. Thus, the molecular basis of the defects which occur in the specific tissues affected in cystinosis need to be studied using differentiated tissue culture systems. Cell cultures closely resembling cystinotic renal proximal tubules (RPTs) are particularly important in this regard, if an understanding of the molecular basis of the renal Fanconi syndrome in cystinosis is to be obtained [1]. Previous studies have indicated that cystinotic RPT cells are more sensitive to oxidative stress and are more likely to undergo apoptosis than normal cells [7]. However, in vitro model systems that more closely resemble cystinotic RPTs are needed to determine the molecular basis for the changes that occur in cystinotic RPTs, as well as how the different *ctns* mutations that cause cystinosis affect renal cells, as well as cells in different organs.

An important means towards achieving this goal is to conduct studies with human-induced pluripotent stem cells (hiPSCs). Not only can hiPSCs be readily derived from human fibroblasts obtained from patients with inherited diseases such as cystinosis [8], but in addition, mutations can be introduced into hiPSCs using CRISPR technology [9]. Moreover, hiPSCs can be differentiated into different types of organoids that resemble tissues present in a number of different organs, including the kidney [10]. Of particular importance, hiPSCs can be differentiated into organoids consisting of RPTs [11]. Thus, impairments in the RPT in the different forms of cystinosis, including infantile cystinosis, can be studied with differentiated hiPSCs.

In the studies described below, hiPSCs were developed from dermal fibroblasts obtained from an individual with the most common mutation in Western Europeans, a 57 kb deletion that enters the *ctns* gene [12,13], in parallel with developing hiPSCs from a “normal” individual. Our studies indicate that cystinotic hiPSCs are pluripotent, although alterations are apparent when normal and cystinotic-derived hiPSCs are differentiated into organoids consisting of RPTs.

## 2. Results

### 2.1. Isolation and Propagation of Normal and Cystinotic hiPSCs

**Figure 1:**
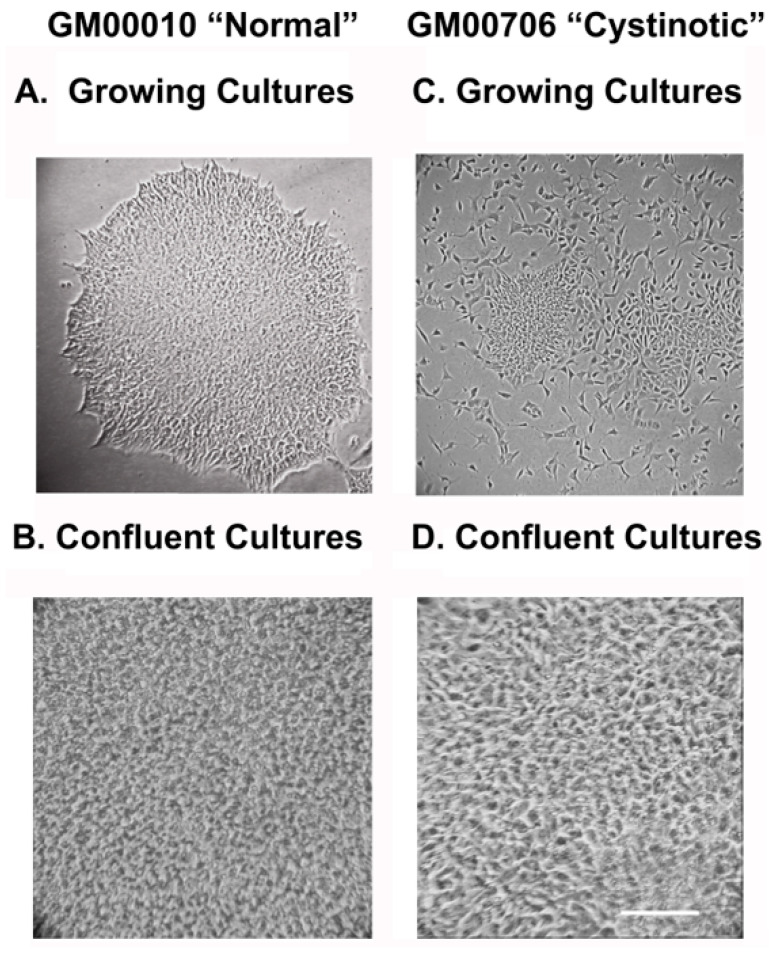
hiPSCs derived from GM00010 and GM00706 Human Fibroblasts. (**A**,**B**) illustrate, respectively, growing and confluent GM00010 “normal” hiPSCs, which (**C**,**D**) illustrate, respectively, growing and confluent GM00706 “cystinotic” hiPSCs. Photomicrographs of hiPSCs under an inverted microscope (100×). Scale bar 50 µm.

Normal and cystinotic human dermal fibroblasts were transfected with three vectors (pXLE-hUL (encoding for L-myc and Lin 28), pCXLE-hSK (encoding forSOX2 and KLF4), and pCXL-hOct3/4-shp53-F) [14]. After replating the cultures onto mouse feeder layers, colonies emerged. Isolated colonies were expanded and grown in E8 medium. Figure 1A shows that hiPSCs derived from “normal” dermal fibroblasts initially formed large, tightly packed colonies at low densities, which eventually grew to form confluent monolayers (Figure 1B). In contrast, hiPSCs derived from cystinotic dermal fibroblasts were more disperse at lower densities (Figure 1C), but nevertheless formed tightly packed monolayers at confluence (Figure 1D), similar to hiPSCs derived from “normal” dermal fibroblasts.

### 2.2. Evidence for the Retention of the 57 kb Deletion in the GM0706-Derived hiPSCs

**Figure 2:**
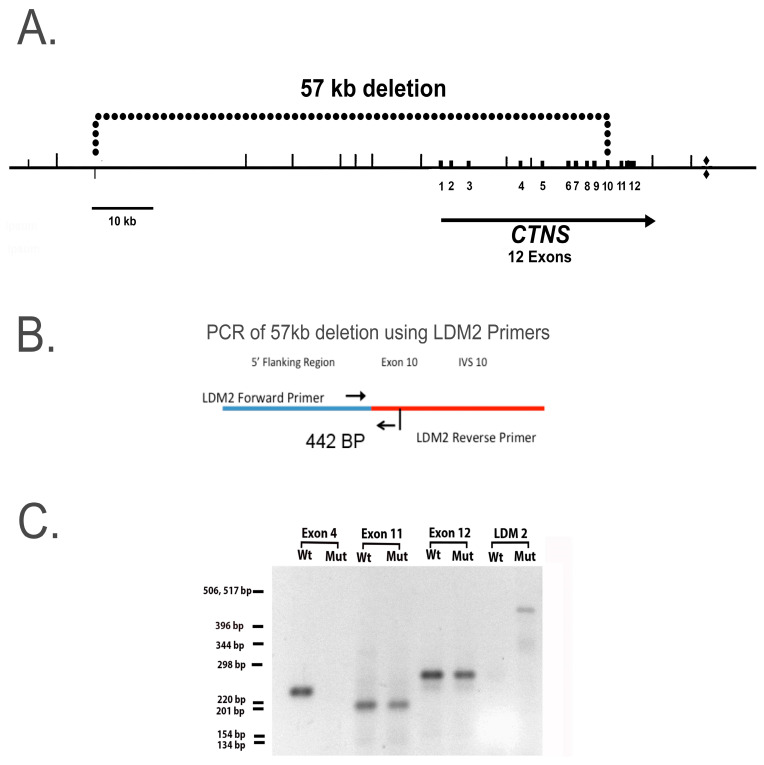
Evidence of the 57 kd deletion in cystinotic hiPSCs. (**A**) Illustration of 57 kb deletion covering part of the *ctns* gene. Numbers 1–12 represent the location of exons 1-12 in *ctns*. (**B**) Illustration of the region covered by LDM2 primers which recognize a region 140 bp upstream from the breakpoint, and a region 362 bp downstream from the breakpoint [13]. The arrows indicate the regions amplified by the forward and reverse LDM2 primers, respectively. (**C**) Amplification of Exons 4, 11, 12, and the region surrounding the terminus of the 57 kb deletion, using LDM2 primers. Genomic DNA was purified from WT and mutant hiPSCs. Subsequently, DNA from exons 4, 11, and 12, as well as from the LDM2 region was amplified by PCR, separated on an agarose gel, and photographed over a transilluminator.

The cystinotic hiPSCs were generated from fibroblasts derived from a cystinotic patient with a 57 kb deletion that extends into the first 10 exons of the *ctns* gene, as shown in Figure 2A. In order to determine whether the hiPSCs derived from the cystinotic fibroblasts retained this deletion, a PCR analysis was conducted. LDM2 primers were employed, which recognize the 5′ and 3′ breakpoints of the 57 kb deletion, as described by Anikster et al. [13] and illustrated in Figure 2B. The results of our PCR analysis are shown in Figure 2C. A 442 BP product was generated from genomic DNA derived from mutant GM0706 hiPSCs (as expected with a 57 kb deletion), unlike the normal GM00010 hiPSCs. In contrast, as shown in Figure 2C, a PCR product was not generated from the genomic DNA of *ctns* mutant GM0076 hiPSCs when using employing primers capable of amplifying exon 4, unlike genomic DNA derived from normal GM00010 hiPSCs. However, PCR products were obtained when amplifying regions in Exon11 and Exon12 in genomic DNA derived from normal GM00010 hiPSCs and mutant GM0076 hiPSCs. These results support the hypothesis that the 57 kb deletion is retained in hiPSCs derived from *ctns* mutant GM0076 fibroblasts.

### 2.3. Pluripotency of the hiPSCs: Expression of ESC Markers

**Figure 3:**
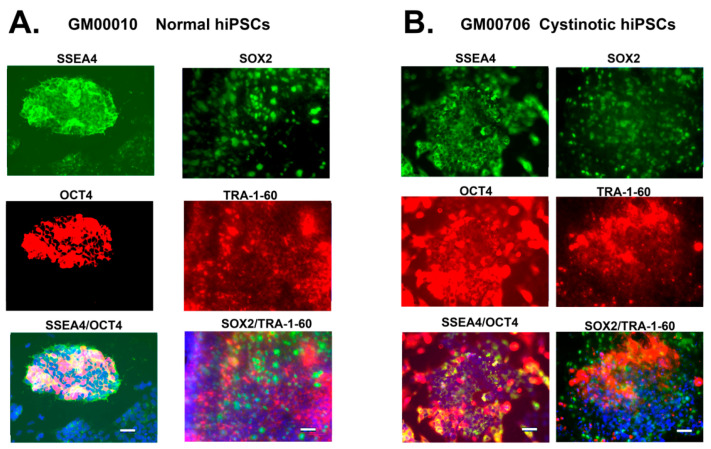
Pluripotent markers of hiPSCs. (**A**) Images of GM00010 normal hiPSCs. (**B**) Images of GM00706 Cystinotic hiPSCs. hiPSCs in SlideFlasks (Nunc) were fixed, permeabilized, and incubated with the primary antibody (rabbit anti-OCT4 and mouse anti-SSEA4, or rat anti-SOX2 and mouse anti-TRA-1). An incubation with secondary antibodies followed (Alex Fluor 555 donkey anti-rabbit (red) and Alexa Fluor 488 goat anti-mouse (green) (OCT4 and SSEA4, respectively), or Alexa Fluor 488 donkey anti-rat (green) and Alexa Fluor 555 goat anti-mouse (red) (SOX2 and TRA-1-60, respectively)). Cells were stained with DAPI and were examined under a Zeiss Axio Observer Fluorescence Microscope at 100×. Scale bars 50 µm.

In order to determine whether the normal and cystinotic hiPSCs are pluripotent, the expression of distinctive Embryonic Stem (ES) cell markers was examined by fluorescence microscopy. The results shown in Figure 3 indicate that the pluripotent markers SSEA4 and Oct4, as well as SOX2 and Tra-1-60 (25), are indeed expressed in cystinotic GM00706, as well as “normal” GM00010 hiPSCs. As a result, these results are consistent with the embryonic (and pluripotent) nature of these hiPSCs.

### 2.4. Pluripotency of hiPSCs: Embryoid Body (EB) Formation

A hallmark of the pluripotency of ES cells, including hiPSCs, is their ability to differentiate into the three primordial germ layers formed during the earliest stages of embryonic development. An in vitro approach used to evaluate whether ES cells can undergo this type of differentiation is to study the ability of ES cells to form embryoid bodies (EBs) consisting of three germ layers (including ectoderm, mesoderm, and endoderm).

**Figure 4:**
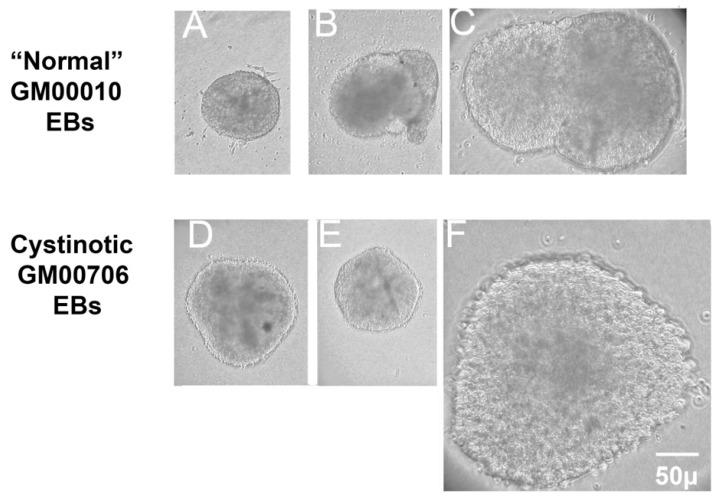
Embryoid Body (EB) Formation. EBs were initiated as described by Lin et al. [3] and were observed under an inverted microscope 5 days later. (**A**–**C**) Normal GM0010 EBs. (**D**–**F**) Cystinotic GM00706 EBs. 100× magnification. 50 µm scale bar.

**Figure 5:**
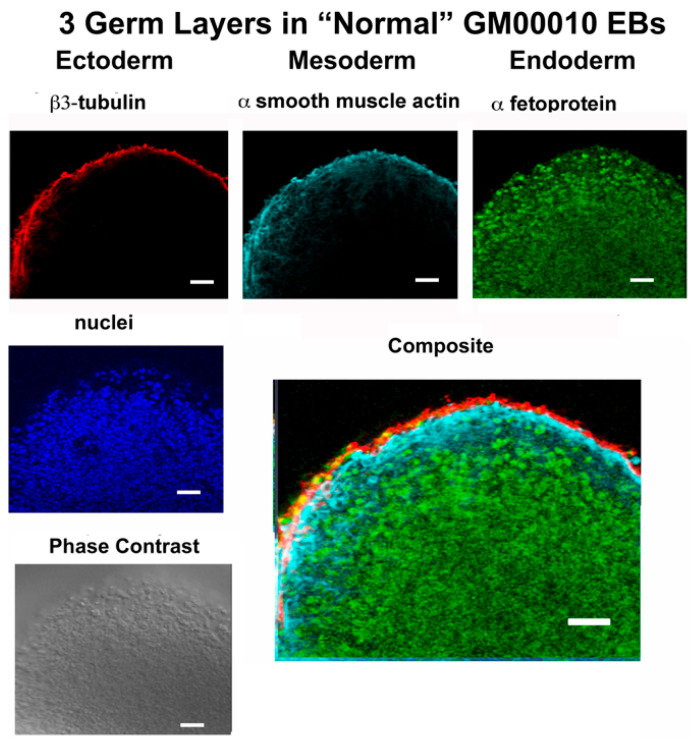
Embryoid bodies possess all three germ layers. Paraffin sections were prepared from EBs derived from normal hiPSCs after 21 days in culture and were examined under a Zeiss Axio Observer Fluorescent Microscope for expression of markers of the three germ layers, including β-3 tubulin (ectoderm), α-smooth muscle actin (mesoderm), and α-1-fetoprotein (endoderm). Markers were observed by staining sections with mouse anti-β-3 tubulin, goat anti-α-smooth muscle actin, and rabbit anti α-1-fetoprotein, followed by an incubation with Donkey anti-mouse Cy3, Donkey anti-goat Cy5 and Donkey anti-rabbit Cy2, and DAPI, as described in Section 4. Scale bars 50 µm.

**Figure 6:**
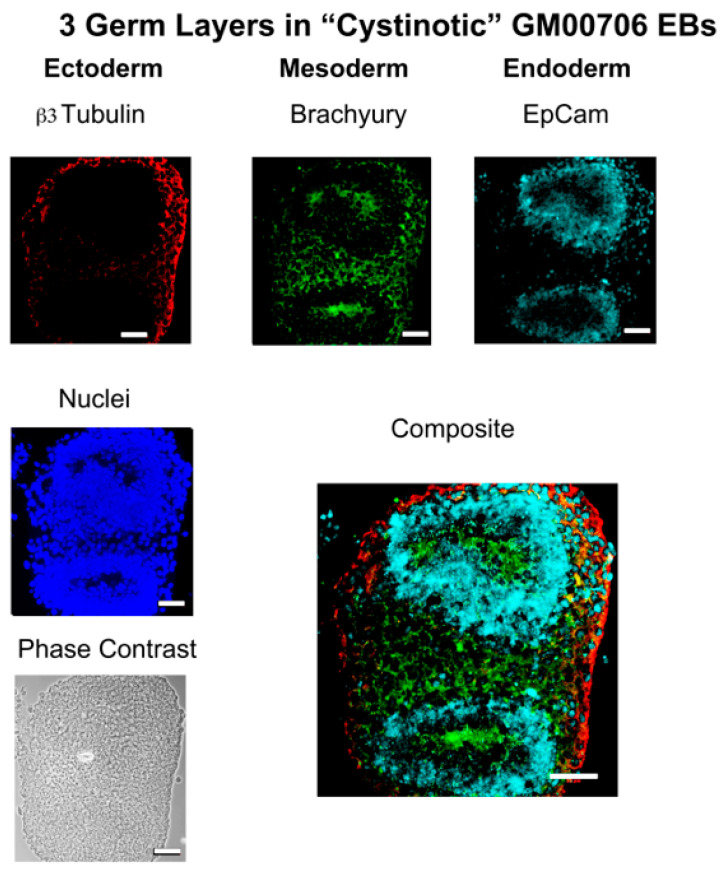
Embryoid bodies derived from Cystinotic hiPSCs. Paraffin sections were prepared from EBs derived from cystinotic hiPSCs after 21 days in culture. The sections were examined under a Zeiss Axio Observer Fluorescent Microscope for the expression of markers of the three germ layers, including β3 tubulin (ectoderm), brachyury (mesoderm), and EpCam (endoderm). Towards these ends, slides were incubated with mouse anti-β3 tubulin, rabbit anti-brachyury, and goat anti-EpCam. Subsequently, slides were incubated with Donkey anti-mouse Cy3, Donkey anti-goat Cy5, Donkey anti-rabbit Cy2, and DAPI, as described in Section 4. Scale bars 50 µm.

Figure 4 shows that EBs were indeed generated from normal (GM00010) and *cystinotic* (i.e., GM00706) hiPSCs. In order to determine whether the EBs possess three germ layers, the EBs were sectioned and examined by fluorescence microscopy (as described in Section 4). Figure 5 shows that appropriate markers, including β3-tubulin, α smooth muscle actin, and α-fetoprotein, were indeed expressed in three distinctive layers (ectoderm, mesoderm, and endoderm, respectively) in an EB derived from normal GM00010 hiPSCs. Similarly, Figure 6 shows that another set of markers, β3-tubulin, brachyury, and EpCaM, were located in three distinctive regions (ectoderm, mesoderm, and endoderm, respectively) in EBs derived from cystinotic GM00706 hiPSCs. Thus, these results further support the hypothesis that both normal and cystinotic hiPSCs (a) are pluripotent and (b) possess the capacity to undergo the initial stages of embryonic development.

### 2.5. Differentiation into Renal Proximal Tubules (RPTs)

Initial symptoms of infantile cystinosis presented themselves during the first year of life in the form of the renal Fanconi Syndrome, characterized by the urinary loss of nutrients and electrolytes. Consequences include dehydration, polydipsia, polyuria, and growth retardation. In infantile cystinosis, the renal Fanconi Syndrome has been attributed, at least in part, to the deterioration of the RPT, presumably due to increased lysosomal cystine. Consistent with this hypothesis, renal tubules of cystinotic infants exhibit the “swan-neck” lesion, characterized by the shortening and narrowing of the neck of the RPT, as well as the eventual dissociation of the RPT from glomeruli. For this reason, it was of interest to determine whether cystinotic hiPSCs can differentiate into RPTs in a manner similar to their normal counterparts.

**Figure 7:**
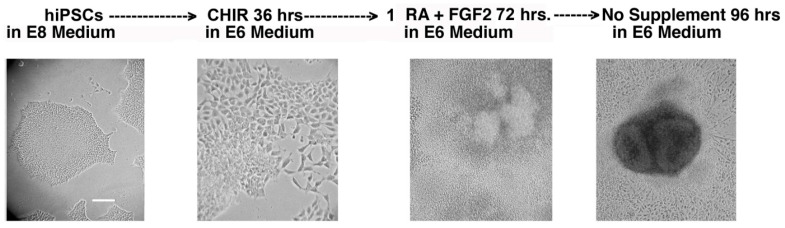
Schema for inducing the differentiation of hiPSCs into RPT Organoids. hiPSCs were cultured in E8 medium prior to their differentiation. The cultures were then incubated for 36 h in E6 medium (i.e., DME/F12 supplemented with insulin and transferrin) containing 5 µM CHIR99021 (a glycogen synthase inhibitor which reportedly causes hiPSCs to differentiate into mesoderm via Wnt signaling) (5). After the 36 h incubation, the cultures were incubated for 72 h in E6 medium containing 1 µM Retinoic Acid (RA) and 250 ng/mL FGF2 (which reportedly stimulates the formation of inner mesoderm (IM) from mesoderm) (5). Subsequently, the cultures were maintained in the E6 medium alone for at least 96 h. Cultures were examined under an inverted microscope during each stage of the differentiation of normal hiPSCs into RPT organoids. Scale bar 50 µm.

The method of Lam et al. [11] was followed to differentiate normal and cystinotic hiPSCs into RPTs, as summarized in Figure 7. After following this protocol, Lam et al. [11] observed that organoids emerged, which expressed several distinctive RPT characteristics, including Lotus tetragonolobus lectin (LTL) binding and the expression of the junctional protein N-cadherin (5).

**Figure 8:**
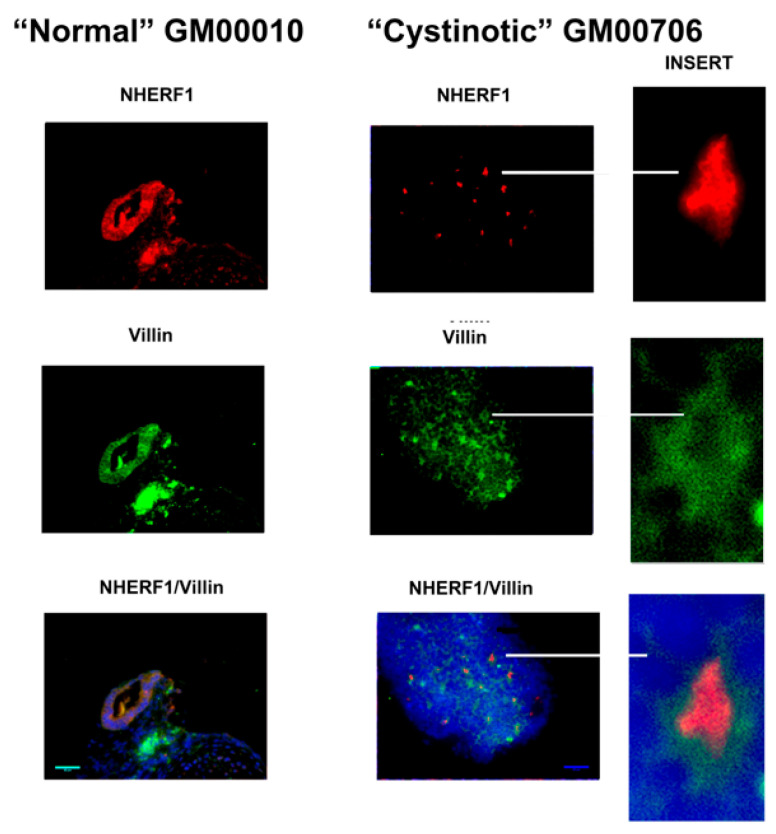
Expression of NHERF1 and Villin in organoids derived from normal and cystinotic hiPSCs. Sections were stained with mouse anti-villin and rabbit anti-NHERF1 antibodies, followed by an incubation with Donkey anti-mouse Cy2 (red) and Donkey anti-rabbit Cy3 (green) secondary antibodies (respectively), treatment with DAPI, and mounting and visualization under a Zeiss Axio Observer. Inserts 10× magnification; scale bar 50 µm.

**Figure 9:**
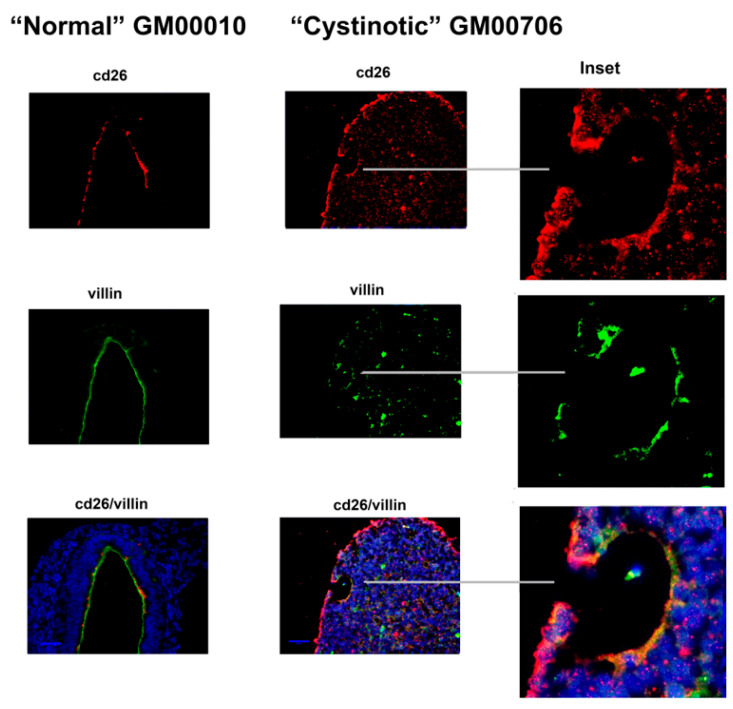
Expression of cd26 and Villin in organoids derived from normal and cystinotic hiPSCs. Sections were stained with rabbit anti-CD26 and mouse anti-villin antibodies, followed by an incubation with Donkey anti-mouse Cy2 (red) and Donkey anti-rabbit Cy3 (green) secondary antibodies (respectively), treatment with DAPI, mounting and visualization under a Zeiss Axio Observer. Scale bar 50 µm. Inserts 5× magnification.

**Figure 10:**
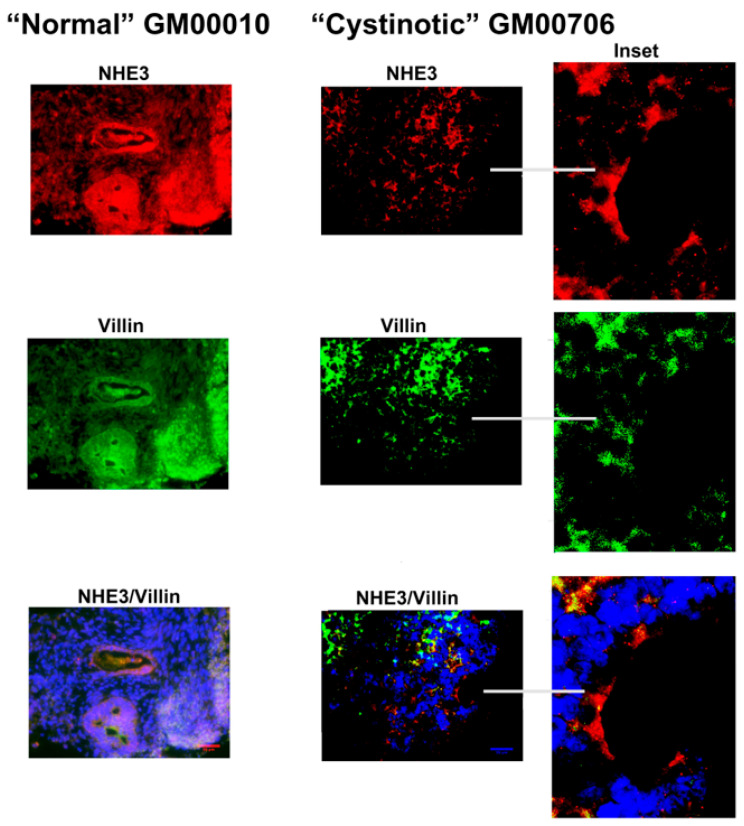
Expression of NHE3 and Villin in organoids derived from normal and cystinotic hiPSCs. Sections were stained with rabbit anti-NHE3 and mouse anti-villin antibodies, followed by an incubation with Donkey anti-mouse Cy2 (red) and Donkey anti-rabbit Cy3 (green) secondary antibodies (respectively), treatment with DAPI, mounting and visualization under a Zeiss Axio Observer. Scale bar 50 µm. Inserts 5× magnification.

Figure 7 illustrates that similar organoids were observed in our hiPSCs following this treatment regime. The organoids were examined by fluorescence microscopy for the expression of distinctive apical markers of the RPT, including cd26 (i.e., dipeptidyl peptidase IV), the Na^+^/H^+^ antiport system isoform 3 (NHE3), the NHE Regulatory Factor 1 (NHERF1), and villin. The results of these studies are illustrated in Figure 8, Figure 9 and Figure 10. Figure 8 shows that tubule-like structures with distinctive lumens were present in cultures derived from normal hiPSCs. Moreover, NHERF1 and Villin were co-expressed in organoids derived from normal hiPSCs. Figure 8 also shows that NHERF1 and villin were also co-expressed in cultures derived from cystinotic hiPSCs, although the structures containing presumptive lumens were much smaller (as indicated in the inserts).

Figure 9 illustrates a large luminal structure present in a differentiated culture derived from “normal” hiPSCs. The lengthy lumen (>200 µm long) observed in cells derived from “normal” hiPSCs was lined with cells co-expressing cd26 and villin. Evidence of the co-expression of cd26 and villin on a luminal surface was similarly obtained in “differentiated” cultures derived from cystinotic hiPSCs (as shown in Figure 9, including Figure 9 inserts). However, much smaller structures with “lumens” were observed in cystinotic hiPSC cultures which had undergone differentiation, as compared with differentiated cultures derived from normal hiPSCs.

Finally, evidence of the expression of NHE3 was obtained in differentiated cultures derived from normal hiPSCs, including a tubule with an intraluminal cast (Figure 10). NHE3 was co-expressed with villin in this tubule, as well as in other structures with developing lumens in this culture. In contrast, the expression of luminal NHE3 and villin was more limited in “differentiated” cultures derived from cystinotic hiPSCs (Figure 10, including Figure 10 inserts).

### 2.6. Response to Chloracetaldehyde (CAA)

The Fanconi Syndrome, which occurs in infantile cystinosis, has been attributed to an increased propensity of cystinotic RPT cells to undergo programmed cell death. According to this hypothesis, the number of cystinotic RPT cells decreases as a consequence of an acceleration in programmed cell death, and this decrease in the number of RPT cells results in a decreased capacity of the RPT to reabsorb solutes, including Na^+^, Pi, and glucose.

In order to evaluate this hypothesis, studies were conducted with CAA, an ifosfamide metabolite that is generated when infants undergo chemotherapy with ifosfamide. CAA has been held responsible for causing the Fanconi Syndrome in such infants. Indeed, our previous studies indicate that CAA is extremely cytotoxic to normal RPT cells in vitro [15]. Thus, it was of interest to determine whether the cytotoxicity of CAA was different in RPTs derived from cystinotic hiPSCs, as compared with RPTs derived from normal hiPSCs.

Both normal GM00010-derived hiPSCs and cystinotic GM00706-derived hiPSCs were differentiated into putative RPTs, as described in Figure 7. The differentiated cultures were then incubated for 6 hr either in the presence or absence of 50 µM CAA. Subsequently, the cultures were stained with SRVAD-FMK FLICA, Sytox Green, and Hoescht 33342 (which, respectively, stain cells undergoing (a) programmed cell death involving caspase activation, (b) dead cells, and (c) the nuclei of all cells).

**Figure 11:**
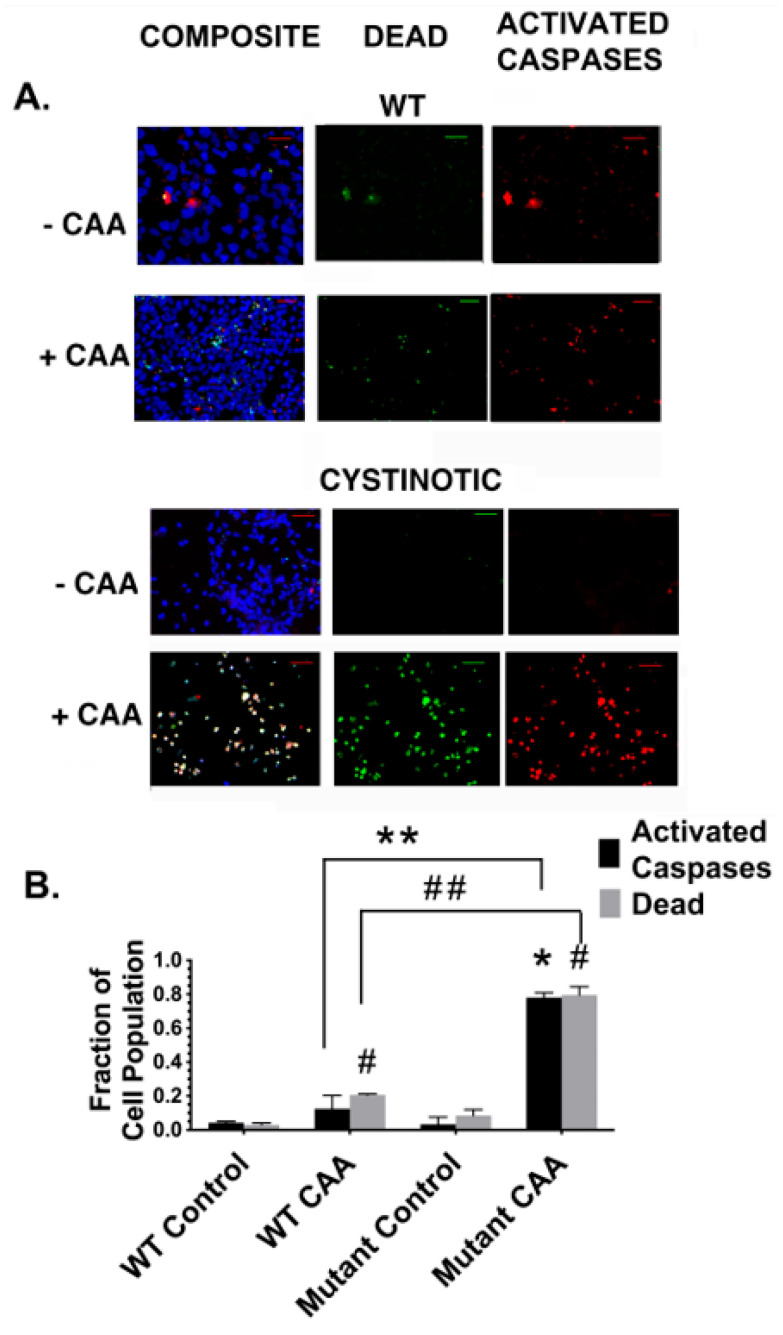
CAA-induced caspase activation in organoids derived from normal and cystinotic hiPSCs. Organoids derived from normal and cystinotic hiPSCs were incubated for 6 h in either the presence of 50 µM CAA (+CAA) or the absence of 50 µM CAA (−CAA). Cultures were stained with SR VAD-FMK FLICA (red), Sytox Green, and Hoechst 33342 to detect cells with activated caspases, dead cells, and the nuclei of all cells, respectively. (**A**) Cultures were examined under a Zeiss Axio Observer Microscope at 100×. Scale bar 50 µm. (**B**) The number of cells stained with these reagents was quantitated using NIH ImageJ in at least 20 microscope fields in each of the three dishes per condition. Values are averages ± SEM of three determinations. Differences between means of (i) ** WT CAA caspase-activated cells vs. Mutant CAA caspase-activated cells, (ii) ## WT CAA dead cells vs. Mutant CAA dead cells, (iii) * Mutant Control vs. Mutant CAA dead cells, as well as (iv) # WT CAA dead cells vs. WT Control dead cells, and Mut CAA dead cells vs. Mutant Control dead cells were considered statistically significant, given that *p* < 0.05.

Figure 11A shows fluorescent images of untreated, control RPT cultures, as well as CAA-treated RPT cultures derived from either normal or cystinotic hiPSCs. The images in Figure 11A indicate that in a large proportion of the cells in cystinotic hiPSC-derived RPT cultures, either caspase activation, and/or cell death had occurred following CAA treatment, unlike RPT cultures derived from normal hiPSCs. The number of apoptotic and dead cells was quantitated using NIH ImageJ version 2.9.0/1.53t. The results shown in Figure 11B indicate that the proportion of cystinotic RPT cells which exhibited activated caspases was 76 ± 4% as compared with only 13 ± 5% in RPT cultures derived from normal hiPSCs. Thus, there was a 6.4 ± 0.7-fold increase in caspase activation in cystinotic-derived “RPTs” following their treatment with CAA as compared to normal RPTs. There was a similar 3.8 ± 0.1-fold increase in dead cells in cystinotic-derived “RPTs” treated with CAA as compared with normal RPTs. These results indicate that RPTs derived from cystinotic hiPSCs exhibit the increased sensitivity to toxicants consistent with our previous report concerning normal RPT cells with reduced expression of cystinosin [16], as well as the observations of Park and Thoene [7]. The increased caspase activation observed in our cystinotic RPT organoids suggests that the increased sensitivity to killing by toxicants in cystinotic RPT organoids can be attributed to apoptosis (which involves Caspase 3 activation), pyroptosis (which involves Caspase 1 activation), and/or the PIDDosome Pathway (which involves the activation of Caspase 2) [17].

## 3. Discussion

In this report, studies have been conducted with hiPSCs derived from the human dermal fibroblasts of an individual with infantile cystinosis, as well as an unaffected individual. Evidence has been presented indicating that both normal and cystinotic hiPSCs are indeed pluripotent. Not only did the normal and cystinotic hiPSCs exhibit distinctive stem cell markers, but in addition both normal and cystinotic derived hiPSCs were able to form EBs containing the three primordial germ layers, which include ectoderm, mesoderm, and endoderm. Thus, these results indicated that our hiPSCs can differentiate into derivatives of all three germ layers.

In order to examine this hypothesis further, our hiPSCs were subjected to a previously described differentiation procedure [11] that has been shown to induce the formation of RPTs. After following this differentiation procedure, our cultures expressed distinctive apical markers of the RPT, including villin, cd26, NHE3, and NHERF1. In differentiated cultures derived from normal hiPSCs, structures resembling tubules with lumens emerged, and moreover, villin, cd26, NHE3, and NHERF1 were located on a surface facing a luminal space. Lumen formation was less pronounced in the “cystinotic” cultures which had undergone “differentiation”, as compared with differentiated cultures derived from normal hiPSCs. The “differentiated” cultures derived from the cystinotic hiPSCs did nevertheless express distinctive RPT markers, including villin, cd26, NHE3, and NHERF.

In addition, following their differentiation into putative RPTs, cultures derived from cystinotic derived cells exhibited an increased sensitivity to killing by CAA when compared to differentiated RPTs derived from normal hiPSCs. The studies with CAA are of particular importance, given that CAA is an ifosfamide metabolite that contributes to the Fanconi Syndrome that often develops in children undergoing a cancer chemotherapy regime that utilizes ifosfamide. The increased sensitivity of the differentiated cells derived from cystinotic hiPSCs to killing by CAA is consistent with previous observations regarding apoptosis in cystinotic RPTs made by Park et al. [7], as well as by our laboratory [16]. Previously, the cytotoxic effects of CAA on a human RPT cell line (i.e., RPTEC) were associated with an increase in intracellular Ca^2+^, while Caspase-3 was not significantly activated, which suggested that necrosis, rather than apoptosis, was involved [18]. However, an increase in intracellular Ca^2+^ has been associated with pyroptosis, a mechanism of programmed cell death which involves Caspase-1 rather than Caspase-3 [19]. Of particular interest in these regards, recently NLRP3 (Nucleotide-binding oligomerization domain, Leucine rich Repeat and Pyrin domain containing 3) was reported as being overexpressed in cystinotic RPT cell cultures [20]. NLRP3 is an integral component of inflammasomes, which are capable of initiating pyroptosis [20]. Moreover, evidence has been presented that the elevated cystine in cystinotic patients not only activates inflammasomes, but additionally activates caspase-1 [21].

In our study, we observed a close correlation between increased caspase activation and cell death in cystinotic RPT organoids treated with CAA, as compared with with normal RPT organoids. Nevertheless, we cannot exclude the possibility that other alterations that have occurred in cystinotic RPT organoids that also contribute to the increase in cell death that occurred in cystinotic RPT organoids treated with CAA. Of particular interest in these regards is autophagy. Autophagy functions to maintain energy metabolism and viability under such conditions as starvation by delivering damaged or potentially harmful cellular components to the lysosomes for digestion and subsequent removal. However, overactivated, autophagy can potentially result in cell death [22]. Indeed, previously, Sansanwal et al. [23] observed an increased number of autophagic vacuoles in cultured human cystinotic RPT cells as compared to normal human RPT cells. In addition, an increased level of markers of autophagosomes was observed, including LC3-II and beclin-1. These authors suggested that increased autophagy contributed to the increased apoptosis observed in their cystinotic cultures. However, Napolitano et al. [24] observed that chaperone-mediated autophagy was defective in their cystinotic RPT cells rather than general macroautophagic flux.

Further studies need to be conducted to resolve these issues. Renal organoids are a powerful tool for this purpose, given that (a) 3-dimensional (3D) culture systems more closely resemble cell in vivo than 2D systems, and (b) they have been derived from hiPSCs, which in turn are readily developed from dermal fibroblasts of individuals with inherited diseases.

In this report, we developed hiPSCs from an individual who is homozygous for a 57-kb deletion which removes the first 9 exons in the *ctns* structural gene, causing the gene product, cystinosin, to be nonfunctional. Studies of RPT cell cultures containing this deletion are particularly important because the 57-kb deletion accounts for approximately 60% of the *ctns* mutations in American patients, and this mutation results in nephropathic cystinosis [13]. The *ctns* gene has been mapped to chromosome 17p13 in 1995; the region has been sequenced and over 140 pathogenic *ctns* mutations have been identified, including the 57-kb deletion, whose sequence is highly conserved [25,26]. Indeed, the 57-kb deletion is thought to have arisen from a single individual in Northern Europe of the first millennium AD [12,13]. Nevertheless, in the absence of a DNA sequence analysis, we cannot exclude the possibility that other mutations have occurred in our human cystinotic RPT cells, although the results of our PCR analysis indicate that the genome of our cystinotic cell cultures is homozygous with respect to the 57-kb deletion.

Organs other than the kidney are eventually affected in infantile cystinosis after the emergence of the renal Fanconi Syndrome [27,28]. These organs include the eye, endocrine organs, the Gastrointestinal tract, muscles, and bones, as well as the central and peripheral nervous system. The other two forms of cystinosis also affect other organs that the kidney, including late-onset juvenile nephropathic cystinosis (where extra-renal dysfunctions observed in infantile cystinosis also develop) as well as non-nephropathic cystinosis, primarily characterized by photophobia. Despite these differences in disease presentation, the three major forms of cystinosis are all the consequence of mutations in the *ctns* gene, which cause alterations in cystinosin function.

The transport functions of the *ctns* gene product, cystinosin, have been studied extensively, including structural studies with three dimensional models [28,29]. Cystinosin, similar to other members of the LCT family, has significant homology with microbial rhodopsins (MRs). Not only do members of the LCT family, including cystinosin, have a similar size to microbial rhodopsins, but in addition these LCTs possess seven highly conservedα -helical transmembrane segments (TMSs) similar to the MRs [2]. The helices within the TMSs undergo large conformational changes during the migration of protons through an access channel [29]. The changes are required because cystine transport by LCTs, including cystinosin, depends upon a proton motive force (pmf) similar to microbial MRs [2]. In the case of cystinosin, the pmf depends upon lysosomal V-ATPase activity. Previous studies indicate that cystine binding to the luminal surface of cystinosin is coupled to proton translocation. The process depends upon two TMSs (including TMSs 1-3 and TMSs 5-7), which are highly conserved regions [2,29], presumably being critical for normal transporter function).

A number of the mutations in the *ctns* gene that result in cystinosis have been observed to alter the cystine transport capabilities of the cystinosin protein [1]. The most prevalent mutation in Northern Europe and North America, the57 kb deletion, w encompasses the promoter and the first 10 exons of *ctns,* as well as two upstream genes (*CARKL* and *TRPV1*) [13]. In addition to the 57 kb deletion, 146 pathogenic *ctns* mutations have been identified within exons 3 through 12, which encode for segments of the structural *ctns* gene [26]. Exons 1 and 2 in *ctns* are noncoding. The mutations include 57 missense and nonsense mutations, as well as insertions and deletions. A number of the missense mutations that have occurred in *ctns* alter the cytoplasmic and luminal gates for protons in the cystinosin protein, as well as the cystine binding site [29]. Of particular interest in this regard, *ctns* mutations in cystinosis often occur within TMSs (particularly in the case of infantile cystinosis). Mutations affecting inter-TMS regions and the N-terminus have been reported (primarily, but not exclusively, in juvenile and atypical cystinosis), including the 2 TMSs involved in proton translocation (i.e.,TMSs 1-3 and TMSs 5-7). Thus, in individuals with such *ctns* mutations, lysosomal cystine transport is impaired, such that intracellular cystine increases in all cells in the body. Nevertheless, such *ctns* mutations primarily have deleterious effects on specific organs, including the kidney. Little is understood about how these mutations cause the functional abnormalities observed in specific tissues in cystinosis. Further studies with cystinotic hiPSCs should prove to be invaluable in these regards.

Initial studies of altered cystine transport by mutated cystinosins led investigators to suggest that the *ctns* mutations that cause infantile cystinosis have more deleterious effects on transport function than the transport alterations caused by *ctns* mutations which result in juvenile or non-nephropathic cystinosis [1]. However, more recent studies of 31 *ctns* mutations (using constructs that translocate to the plasma membrane) have indicated that some *ctns* mutations that cause infantile cystinosis (including *ctns* S298N and *ctns* W182R) permit normal cystine transport activity to be retained, whereas several mutations causing juvenile cystinosis (including *ctns* N323K and *ctns* K280R) result in a complete loss of cystine transport activity [1]. These observations are not consistent with the hypothesis that lysosomal cystine accumulation is aalswaysthe primary pathogenic cause of cystinosis.

Of particular interest in these regards are recent studies indicating that cystinosin (similar to other LCTs and MRs) has other functions in addition to transport. One such function involves the regulation of Chaperone Mediated Autophagy (CMA) due to the role played by cystinosin in trafficking the CMA receptor (i.e., LAMP2A) [30], while another such function is the regulation of the mammalian Target of Rapamycin Complex 1 (mTORC1). Recent studies indicate that cystinosin is a component of the V-ATPase-Ragulator-Rag complex, that controls mTORC1 activation [31,32]. The interaction of the V-ATPase-Ragulator-Rag complex, including cystinosin, with mTORC1 promotes mTORC1 activation, an event which stimulates anabolic programs. Of particular interest in these regards is the recent report by Berquez et al. [32]—that the lysosomal cystine storage that occurs in cystinosis stimulates the Ragulator-Rag GTPase-dependent recruitment of mTORC1, resulting in its constitutive activation. The studies conducted by Berquez et al. [32] also suggest that the constitutive activation of mTORC1 caused by lysosomal cystine storage diverts RPT cells towards growth and proliferation so as to disrupt the expression of their differentiated functions. The recent studies conducted by Luciani and Devuyst [33] further supports this hypothesis, and further suggests that lysosomal cystine accumulation actually causes an impairment of cell fate decisions by presumptive RPT cells in the kidney.

In the studies reported here, cystinotic hiPSCs that were differentiated into RPTs were less likely to form tubules with lumens than normal hiPSCs which had been differentiated into RPTs. This observation is consistent with the studies of Berquez et al. [32], as well as Luciani and Devuyst [33], whose studies indicated that lysosomal cystine storage resulting from the loss of functional cystinosin negatively affects cell fate specialization, causing a change in RPT cells so that they are no longer in a catabolic state, which promotes differentiation, but instead are in an anabolic state that promotes growth and proliferation. According to these investigators, this change is a consequence of the constitutive activation of mTORC1 signaling in *CTNS*-deficient RPT cells. These investigators propose that these changes were not observed in RPT cell line derived from cystinotic patients, presumably due to the immortalization process. However, in this report, our cystinotic and normal hiPSCs were derived using episomal vectors which were only expressed temporally and thus did not alter gene expression over the long term. Further studies are needed to examine effects of the mTORC1 complex on our cystinotic hiPSCs and their differentiation into RPTs.

## 4. Materials and Methods

### 4.1. Materials

Dulbecco’s Modified Eagle’s Medium (DME), Ham’s F12 Medium (F12), fetal bovine serum, growth factor deficient matrigel, the 1KB plus DNA Ladder, and the Image-IT LIVE Red Poly Caspase Detection Kit and SlideFlasks were from ThermoFisher (Waltham, MA, USA). Human insulin, human holo-transferrin, 2-phosphoascorbate, penicillin and streptomycin, and other chemicals were from Sigma-Aldrich Chemical Corp. (St. Louis, MO, USA). Selenium was from Difco laboratories (Detroit, MI, USA). bFGF and TGFb1 were from Novoprotein (Summit, NJ, USA). Secondary antibodies were from Jackson ImmunoResearch Laboratories, Inc. (West Grove, PA, USA). Plasmids, including pCXLE-hUL, pCXLE-hSK, and pCXLE-hOct3/4-shp53-F, were from Addgene (Watertown, MA, USA), having been deposited by Shinya Yamanaka.

### 4.2. Generation of hiPSCs

Integration-free human iPSCs (hiPSCs) were generated from human dermal fibroblasts homozygous for the 57 kb deletion in *ctns*, which results in nephropathic cystinosis (obtained from the Coriell Institute for Medical Research (Camden, NJ, USA), Cat. # GM00706), as well as “normal” human fibroblasts (Coriell # GM00010), used as controls in the studies of GM00706 by Park and Thoene [7]. The fibroblasts were maintained in Dulbecco’s Modified Eagle’s Medium (DME) supplemented with 20 mM sodium bicarbonate, 10% fetal bovine serum (FBS), 0.5% penicillin, and streptomycin.

**Figure 12:**
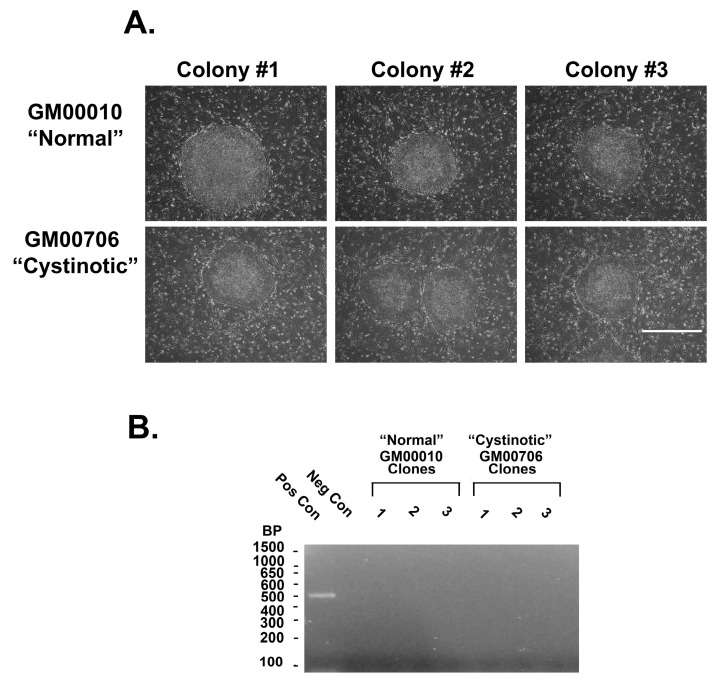
Colonies of normal and cystinotic hiPSCs. (**A**) Photomicrographs were observed under an inverted microscope (100×) 50 µm scale bar. (**B**) Absence of EBNA1 in hiPSCs. EBNA1 was PCR amplified from three clones of GM00010 and GM00706 (as well as positive and negative controls), followed by agarose gel electrophoresis and band detection.

Three vectors were introduced by electroporation into the cystinotic and normal human fibroblasts, including (1) pCXLE-hUL encoding for L-myc, and Lin28, (2) pCXLE-hSK encoding for SOX2 and KLF4, as well as (3) pCXLE-hOct3/4-shp53-F, encoding for Oct 3/4 and shRNA against human p53, as described by Okita et al. [14]. The vectors also encoded for Epstein-Barr virus nuclear antigen 1 (EBNA1). Seven days later, transfected cells were replated into culture dishes coated with mouse feeder layers, as described by Takahashi et al. [34]. Two weeks later, colonies and ESC-like morphology emerged, both from the electroporated normal (GM00010) (Figure 12A) and cystinotic fibroblasts (GM00706) (Figure 12A). Colonies were isolated and expanded, as described by Takahashi et al. [34].

Previously, Yu et al. [35] found that when using episomal vectors to obtain pluripotent hiPSCs, the vectors were spontaneously lost. Despite this, the cultures retained a stable pluripotent state. In order to determine whether the vectors were similarly lost from our hiPSCs, the presence of EBNA1 in episomal DNA was examined in individual clonal isolates of GM00010 and GM00706 hiPSCs after 3 months in cultures, as well as from positive control cultures (cultures obtained 2 weeks after transfection of dermal fibroblasts with the 3 vectors) and untransfected cultures (negative controls). A PCR analysis of EBNA1 in episomal DNA was conducted using forward and reverse primers (ATCGTCAAAGCTGCACACAG and CCCAGGAGTCCCAGTAGTCA, respectively), as described by Yu et al. [35]. Figure 12B shows the absence of EBNA1 3 months after transfection (unlike the positive control), consistent with the loss of the original episomal vectors.

### 4.3. PCR Analysis of CTNS

Genomic DNA was purified from normal and cystinotic hiPSCs using a Monarch Genomic DNA Purification Kit (NEB #T3010) and PCR amplifications conducted using a Bio-Rad CFX96 Touch qPCR System (Bio-Rad Laboratories, Hercules, CA, USA.) as described by Anikster et al. [13]. Primers employed to amplify specific regions in the *ctns* gene included (a) LDM2 primers (Forward, 5′-ACCTCTCTGATGTGTCCAAG-3′, Reverse, 5′-AGCCAAAGGCATCAGGAAAG-3′), which amplify the breakpoint in the 57 kb deletion (illustrated in Figure 3B, and described by Anikster et al. [13]), as well as (b) primers that amplify specific exon regions in *ctns* as described by Town et al. [25], including primers for exon 4 (forward 5′-GTCATTGATTTGGGTCC-3′; reverse 5′-TAGGGCTTGTCTTACAGGTA-3′), exon 10 (forward 5′-GGCCTCTGTGTGGGTCC-3′; reverse 5′-GGCCATGTAGCTCTCACCTC-3′), and exon 11 (forward 5′-GCCCTCCGTCTGTATGTCCG-3′; reverse 5′-GCCCGATGCCCCAGC-3′). PCR products were separated on agarose gels and visualized on a Fotodyne Photo Prep UV transilluminator Model 3-3500 (Fotodyne, Inc., Hartland, WI, USA).

### 4.4. Culture of hiPSCs

Both normal and cystinotic hiPSCs were cultured in chemically defined E8 medium on matrigel coated dishes in a 5% CO_2_/95% air humidified environment at 37 °C, as described by Chen et al. [36]. E8 medium was prepared from a basal medium (DME/F12) which consisted of a 1:1 mixture of Dulbecco’s Modified Eagle’s Medium and Ham’s F12 containing a total of 20 mM sodium bicarbonate, 118 mM NaCl, penicillin (92 unit/mL), and streptomycin (200 µg/mL). E8 medium was obtained by further supplementing DME/F12 with 20 µg/mL human insulin, 10 µg/mL human holo-transferrin, 100 µg/mL FGF2, 2 ng/mL TGFβ, 64 µg/mL 2-phospho-L-ascorbate, and 5 × 10^−8^ M selenium.

The medium was changed daily until the cultures became confluent. Confluent hiPSC cultures were passaged by first incubating the hiPSCs for 5 min at 37 °C in Phosphate Buffered Saline (PBS) supplemented with 0.5 mM EDTA (EDTA/PBS solution), followed by centrifugation (in EDTA/PBS) and resuspension of the cultures in E8 medium supplemented with Rock Inhibitor Y27632 (10 µM). Cultures were then plated into culture dishes coated with matrigel, as detailed by Lin and Chen [37].

### 4.5. Pluripotency Detection: Identification of Stem Cell Markers

Immunostaining of hiPSCs was conducted using SlideFlasks, as described by Marti et al. [38]. To summarize, the medium was removed from the cultures in the SlideFlasks prior to microscopy, and cultures were fixed with 4% *w*/*v* p-formaldehyde. The cultures were blocked (with TBS + 0.5% *v*/*v* Triton X-100 + 6% *v*/*v* donkey serum), followed by incubation with primary antibodies in TBS++ (i.e., TBS + 0.1% *v*/*v* Triton X-100 and 3% donkey serum). Antibody combinations used included (A) rabbit anti-Oct4 (A24867 ThermoFisher, Waltham, MA, USA) and mouse anti-SSEA4 (A24866 ThermoFisher), as well as (B) rat anti-SOX2 (A24759, ThermoFisher) and mouse anti-TRA-1-60 (A24868, ThermoFisher). After the incubation with primary antibodies, the cultures were washed 3 times with TBS++ and incubated 2 h at 37 °C with secondary antibodies in TBS++ (including AlexaFluor 594 donkey anti-rabbit and AlexaFluor 488 goat anti-mouse IgG3 in the case of combination A, as well as Alexa Fluor 488 donkey anti-rat and Alexa Fluor 595 goat anti-mouse IgM in the case of combination B). After washing 3 times with TBS, the slides were incubated with DAPI, mounted, and examined under a Zeiss Axio Observer Fluorescent Microscope at 100×.

### 4.6. Pluripotency Detection: Formation of Embryoid Bodies (EBs) with Three Germ Layers

EBs were generated from hiPSCs that were 60–80% confluent, as described by Lin and Chen [37]. After washing the cultures twice with EDTA/PBS, the cells were incubated with EDTA/PBS for 10 min. Aggregates were disrupted by pipetting and transferred to a 15 mL tube containing an equal amount of E8 medium containing 5 mg/mL Polyvinyl Alcohol (PVA) (E8/PVA medium) and 110 µM Rock Inhibitor Y-27632. The cells were spun at 10,000 rpm 5 min, followed by resuspension in EB/PVA medium with 110 µM Y-27632 at 10^5^ cells/mL (determined using a Coulter Counter Model Zf, Beckman Coulter, Chasman, MN, USA. To form EBs, 20 µL drops were hung on the lid of Petri dishes and were incubated for two days. The cultures were then transferred into Corning low attachment dishes for further culturing.

Subsequently, the EBs were collected and placed into a small drop of molten 2% Low Melting Point (LMP) Agarose (ThermoFisher, Waltham, MA, USA) in PBS and cooled, as described by Shamblott et al. [39]. The EBs in the solidified LMP Agarose were fixed with 3% p-formaldehyde in PBS and embedded in paraffin. Sections (6 mm) were placed upon Probe on plus microscope slides (Fisher Sci., Hampton, NH, USA).

Sections to be used for immunostaining were dewaxed and rehydrated by serial incubation with xylene and decreasing gradations of ethanol. For antigen retrieval, the slides were incubated for 20 min at 95 °C in 10 mM Tris, pH 9.0, 1 mM EDTA, and 0.05% Tween 20. The slides were then incubated with TBS, followed by 100 mM glycine to minimize autofluorescence, permeabilized with 0.25% Triton X-100 in TBS, and blocked with TBS containing 0.25% Triton X-100 and 3% donkey serum.

Sections were then incubated overnight at 4 °C with primary antibody in TBS++. Antibody combinations included (A) mouse antiβ-tubulin (Clone TUJ1, R&D Systems, MAB1195; ectoderm), goat antiα-smooth muscle actin (R&D Systems, NB300-978; mesoderm), and rabbit anti-α-1-fetoprotein (Dako A0008; endoderm), as well as (B) mouse anti-β3-tubulin (Clone TUJ1, R&D Systems, MAB1195; ectoderm), rabbit anti-brachyury (R&D Systems MAP20851; mesoderm), and goat anti-EpCam (R&D Systems AF960; endoderm). Following the primary antibody incubation, slides were washed and incubated with secondary antibodies in TBS++ for 2 h at 37 °C. Included amongst the secondary antibodies utilized were Donkey anti-mouse Cy3, Donkey anti-goat Cy5, and Donkey anti-rabbit Cy2 in the case of antibody combination A, as well as Donkey anti-mouse Cy3, Donkey anti-rabbit Cy2, and Donkey anti-goat Cy5. After the incubation with secondary antibodies, slides were treated with DAPI, mounted, and sections were visualized under a Zeiss Axio Observer Fluorescent Microscope at 100×.

### 4.7. Differentiation of hiPSCs into Renal Organoids

The method of Lam et al. [11] was employed to differentiate normal and cystinotic hiPSCs into RPTs. To summarize, (a) the hiPSCs were incubated for 36 h in E6 medium (i.e., DME/F12 supplemented 20 µg/mL human insulin and 10 µg/mL human transferrin) and were further supplemented with 5 µM CHIR99021, a glycogen synthase inhibitor (which induces the differentiation of hiPSCs into mesoderm via the Wnt pathway (5)). Subsequently, (b) the cultures were incubated for 72 h in E6 medium supplemented with 100 ng/mL FGF2 and 1 µM retinoic acid (which induces the formation of inner mesoderm (IM) from mesoderm) (5). Finally, (c) the cultures were maintained for ≥7 days in E6 medium with no further supplements, resulting in the emergence of organoids which expressed distinctive RPT characteristics, including Lotus Tetragonolobus Lectin binding and junctional N-cadherin (5).

The renal organoids that appeared after 7 days were removed from culture dishes with a rubber policeman, transferred to a 15 mL tube, and were centrifuged at 500 rpm. The culture medium was removed and replaced with PBS containing 3.7% p-formaldehyde (PFA). After an overnight incubation, the cultures were washed by centrifuged to remove the PBS + PFA, followed by 2 washes with PBS. The tube containing the hiPSCs was put into a 37 °C waterbath. After adding molten 2% low melting point agarose in PBS to the tube, the hiPSCs in the agarose were transferred to parafilm, where the agarose solidified. The hiPSCs in the agarose were embedded in paraffin, and 6 mm sections were transferred onto Probe on Plus microscope slides.

The sections were dewaxed, rehydrated, and immunostained, as described above with EBs. Antibodies combinations employed in immunostaining included (A) rabbit polyclonal anti-cd26 (sc-9153, H-270, Santa Cruz Biotech, Dallas, TA, USA) and mouse monoclonal anti-villin (sc-58897, Santa Cruz Biotech), (B) rabbit polyclonal anti-NHERF-1 (sc-134485, H-100, Santa Cruz Biotech) and mouse monoclonal anti-villin (sc-58897, Santa Cruz Biotech), as well as (C) rabbit polyclonal anti-NHE3 (sc-28757, H-170, Santa Cruz Biotech) and mouse monoclonal anti-villin (sc-58897, Santa Cruz Biotech). Subsequently, slides were stained with a Donkey anti-mouse Cy2 secondary antibody and a Donkey anti-rabbit Cy3 secondary antibody. Slides were treated with DAPI and were mounted and visualized under a Zeiss Axio Observer Fluorescent Microscope (100×).

### 4.8. Quantitation of Apoptosis and Cell Death

The frequency of apoptotic and dead cells was determined using an Image-IT LIVE Red Poly Caspase Detection Kit. To summarize, apoptotic cells were identified using a Fluorescent Labelled Inhibitor of Caspases (FLICA). SRVAD-FMK FLICA reagent (that recognizes activated caspase 1, as well as caspases 3-9, with red fluorescence). To detect dead cells, the cultures were co-stained with Sytox Green (a nuclear stain that permeates cells with compromised membranes, typical of dead cells). Hoechst 33342 was employed to detect nuclei (via blue fluorescence). Cultures were examined under a Zeiss Axio Observer Fluorescence Microscope (Zeiss, Oberkochen, Germany) at 100×. The number of cells stained with these reagents was quantitated using NIH ImageJ in at least 20 microscope fields in each of the three dishes per condition.

### 4.9. Statistical Analysis

Statistical analyses were conducted using GraphPad Prism Version 10.0.3. Statistical results were expressed as Means ± SEM. Statistical differences between groups were determined by means of a Chi-square (and Fisher’s exact) test, also using GraphPad Prism. Differences between means were considered statistically significant when *p* < 0.05.

## Data Availability

The data presented in this study are available on request from the corresponding author.
